# Pharmacogenomics in drug therapy: global regulatory guidelines for managing high-risk drug reactions

**DOI:** 10.1038/s41431-025-01950-6

**Published:** 2025-09-24

**Authors:** Safa Omran, Siew Hua Gan, Siew Li Teoh

**Affiliations:** 1 School of Pharmacy, Monash University Malaysia, Jalan Lagoon Selatan, Bandar Sunway, Subang Jaya, Selangor Malaysia; 2 Faculty of Pharmacy, University of Cyberjaya, Persiaran Bestari, Cyber 11, Cyberjaya, Selangor Malaysia

**Keywords:** Outcomes research, Drug regulation

## Abstract

Pharmacogenomics is rapidly transforming precision medicine, yet regulatory policies governing its implementation vary widely across countries. This review aims to provide a global perspective on pharmacogenomics guidelines, with a particular focus on high-risk drug reactions such as carbamazepine therapy-induced severe cutaneous adverse reactions. Carbamazepine was selected as a representative example due to its inclusion on the World Health Organization’s essential medicines list and its well-documented association with high-risk alleles, which are linked to severe cutaneous adverse reactions such as Stevens-Johnson Syndrome and Toxic Epidermal Necrolysis—conditions with significant mortality rates. Two databases, Overton and Dimensions, were searched to identify relevant national guidelines and policy documents in English. Countries were identified based on document availability and access to governmental sources. The review revealed that all examined countries recognized genetic variation in carbamazepine response within their guidelines, showing notable consistency. However, religious implications related to pharmacogenomics were largely absent. The findings also indicated a growing global momentum toward integrating pharmacogenomics into healthcare systems, although the depth and scope of regulation differ. The United States stands out for its comprehensive pharmacogenomics policy framework, which extends to clinical and industry settings. Lessons from the U.S. model can inform policy development in other regions, tailored to each country’s healthcare infrastructure and cultural context. In conclusion, global harmonization of pharmacogenomics policies is essential to foster international collaboration, enable data sharing, and enhance the safe and equitable implementation of pharmacogenomics in clinical practice.

## Introduction

“One drug does not fit all” was the impetus for developing precision medicine, of which pharmacogenomics (PGx) is a vital component [1]. The field of PGx is undergoing rapid advancement, giving rise to various ethical, legal, social (ELS), religious, economic, and clinical concerns that can potentially influence its overall implementation and patient outcomes. In order to effectively engage PGx practice with the ultimate goal of improving health outcomes, the domain of ethical, legal, and social consequences is a crucial component of its competency, as outlined by the American Association of Colleges of Pharmacies (AACP) [2]. For example, implementing PGx may cause the inadvertent disclosure of an individual’s disease or medical condition, potentially leading to discrimination, stigmatization, and ineligibility for employment and insurance coverage [3]. In fact, since the rapid evolution of the PGx field, policies and regulations pertaining to PGx and its corresponding concerns and implications may exhibit substantial variation across countries, contingent upon their respective degrees of progress in this area.

Worldwide, as compared to Asian countries and even Australia as well as Canada, countries in northern American and European countries largely have a greater abundance of genomic and PGx publications, projects, and more well-established frameworks_._ To date, although some countries have made progress in translating PGx research into clinical practice, all countries must overcome similar challenges and barriers in implementing PGx [4]. Additionally, countries in Southeast Asia tend to exhibit a significant degree of ethnic diversity, which may affect the variability seen in genetic and pharmacological responses among the said population. Interestingly, in recent years, there has been a noticeable increase in the number of research publications focused on PGx within these countries, with special emphasis on some fields, including central nervous system disorders, cancer, and blood-related conditions [5].

One of the most important findings from Asian-specific research is the studies that reported cases of drug-induced severe cutaneous adverse reactions (SCARs), such as Stevens-Johnson syndrome and Toxic Epidermal Necrolysis (SJS/TENS). Although SJS/TEN may be rare, it requires hospital admission, and even following recovery, patients may still experience serious consequences such as vision loss, thus placing an economic burden on the healthcare system. The primary suspected drug classes included antiepileptic and antipsychotic agents such as carbamazepine, phenytoin, oxcarbazepine, and lamotrigine, along with antibiotics/antiviral drugs and allopurinol [5].

Medications that induce SJS/TEN are particularly associated with high-risk alleles, notably human leukocyte antigen (*HLA*) alleles, such as *HLA-A* and *HLA-B* [6]. These genes are among the most highly polymorphic in the human genome [7]. For example, HLA- B*15:02 is strongly associated with carbamazepine (CBZ)- induced SJS/TEN [8]. CBZ is one of the commonly used aromatic antiepileptics that is also indicated for many other medical conditions [7]. Although HLA-B*15:02 is not a universal marker since its prevalence is low in Europe, Japan, Korea, Sri Lanka, and the majority of ethnic groups in India, its prevalence is rather high in some Asian ethnicities. For example, reports from Asia indicate that the prevalence of HLA-B*15:02 is: (1) 5–15% among the Han Chinese in Taiwan, Hong Kong, Malaysia, and Singapore, (2) 12–15% among Malays in Malaysia and Singapore, and (3) 8–27% among Thais. However, it is (1) less than 10% among Vietnamese [9] and (2) predominantly absent in individuals not of Asian origin (e.g., Caucasians, African Americans, Hispanics, and Native Americans) [8].

HLA-A*3101 allele is another example that is moderately associated with the development of hypersensitivity reactions, including SJS/TEN, in patients who are using CBZ. The prevalence of HLA-A*3101 is anticipated to be greater than 15% among patients of Japanese, Native American, Southern Indian (e.g., Tamil Nadu), and some Arabic ancestry; up to approximately 10% among patients of Han Chinese, Korean, European, Latin American, and other Indian ancestry; and up to approximately 5% among African Americans [10].

Significantly, in patients with positive HLA-B*15:02, it was found that other aromatic anti-epileptics with similar chemical structures to CBZ, including oxcarbazepine, phenytoin, fosphenytoin, and lamotrigine, may have cross-reactivity and a greater risk of hypersensitivity reactions, including SJS/TEN [6]. Therefore, screening patients for *HLA* alleles, particularly HLA-B*15:02, prior to initiating CBZ therapy may reduce the incidence and risk of CBZ-induced SJS-TEN and optimize treatment safety and efficacy in some populations.

Overall, the lack of standardized policies/guidelines governing PGx practice constitutes a major barrier to adopting, implementing, and providing PGx services in the healthcare system [4]. In this review, we aim to highlight and provide insights into the global status of policies and guidelines that regulate the PGx practice and its related concerns. In particular, the review compares the current practice policies and guidelines in the context of CBZ therapy. CBZ is selected as an example of medication for PGx-related clinical concerns since it is one of the essential medications listed by the World Health Organization (WHO) [11] and is strongly associated with high-risk *HLA* alleles, cross-reactivity with other medications [6], and the prevalence of induced SJS/TEN [9], which has significant mortality rates of up to 10% (SJS) and 50% (TEN) [8].

## Materials and methods

Key countries were identified based on the following criteria: the availability of documents in English as well as accessibility to the relevant governmental organizations. Guidelines governing CBZ therapy were examined and compared across countries. The policy review strategy included a specific search of two electronic databases: Overton and Dimensions. Additionally, the websites of relevant governmental organizations were searched. A broad search strategy was employed using three research concepts: PGx, clinical implementation, and policy. A range of search terms related to the research concepts was combined with appropriate Boolean operators. The keywords were (pharmacogenomics OR pharmacogenetics OR genomics OR genetics) AND (practice OR implementation OR application) AND (policy OR guideline OR guidance document OR law OR regulation OR position statement).

All the essential information for the review was extracted from the included papers and relevant websites, including the name of the authorized healthcare organization for each country, PGx settings, such as clinical, research, and industry, PGx drug labeling, the governmental standards for PGx testing laboratories, PGx testing reimbursement and insurance coverage, PGx patient data, including informed consent, privacy, and confidentiality, and the availability of a non-genetic discrimination act that prohibits discrimination based on genetic information with respect to health insurance and employment. The extracted data were classified into the current general regulations of PGx practice and in the context of CBZ therapy in each country. A qualitative synthesis and narrative summary with tabled results were used to illustrate how the findings relate to the review’s aims.

## Results

The current general regulations regarding PGx practice in each country are outlined in Table 1.

**Table 1:** Global status of policies regulating PGx practice.

Country		Regulatory Authority	Research Consortium	PGx Settings ^†^	PGx Drug Labeling	PGx Testing Laboratory ^‡^	PGx Testing Reimbursement ^§^	Genetic Non-Discrimination ^¥^	PGx Patient Data ^œ^
**Africa**	Egypt	Egyptian MOH & P	–	Clinical & research	NK	NK	I	NK	A
	Kenya	Kenya MOH	–	NK	NK	NK	NK	NK	A
	Mauritius	MOH and Quality of Life	–	NK	NK	NK	NK	NK	A
	South Africa	DH (South Africa)	–	NK	NK	NK	NK	NK	A
**Asia**	Hong Kong	DH (Hong Kong)	–	NK	NK	NK	A	A	A
	Japan	PMDA	–	NK	A	NK	A	NK	NK
	Malaysia	Malaysia MOH	–	Clinical & research	NK	NK	NK	I	A
	Singapore	Singapore MOH	–	Clinical & research	NK	NK	A	NK	I
	Taiwan	MOHW	–	Clinical & research	NK	NK	A	NK	NK
	Thailand	Thailand MOH	–	Clinical & research	NK	NK	A	NK	NK
**Australia**		Department of Health & Aged Care	–	Clinical & research	A	A	NK	NK	NK
**Canada**		HC	CPNDS	Clinical & research	A	NK	NK	NK	NK
**Europe**	European Union	EMA	U-PGx	Clinical & research	A	NK	NK	NK	A
	Netherlands		DPWG	Clinical & research	A	NK	A	NK	NK
**UK**		NHS	–	Clinical & research	A	NK	A	NK	NK
**US**		FDA	–	Clinical, research & industry	A	A	A	A	A

Keys: NK: Not Known/Accessible to authors. A: Accessible/Available.

^†^: PGx settings are categorized into clinical, research, and industry settings.

^‡^: PGx testing laboratory government standard.

^§^: PGx testing reimbursement and insurance coverage.

^¥^: Act which prohibits discrimination based on genetic information with respect to health insurance and employment.

^œ^: Patient genetic data protection (informed consent, privacy, and confidentiality).

^**–:**^ No information is available/applicable.

^I^: Initiative proposed by the government.

*CPNDS* Canadian Pharmacogenomics Network for Drug Safety, *DH* Department of Health, *DPWG* Dutch Pharmacogenetics Working Group, *EMA* European Medicines Institution, *FDA* the US Food and Drug Administration, *HC* Health Canada, *MOH* Ministry of Health, *MOH&P* Ministry of Health and Population, *MOHW* Taiwanese Ministry of Health and Welfare, *NHS* National Health Service, *PMDA* Pharmaceuticals and Medical Devices Agency, *U-PGx* European Ubiquitous Pharmacogenetics Consortium.

### Existing regulations on PGx practice globally

In the context of healthcare service policy, the WHO established a Technical Advisory Group on Genomics in light of the fifteen recommendations put forth by the Science Council, an entity that the WHO founded in 2021. The recommendations are organized into four distinct categories: promotion of genomics through advocacy, implementation of genomic methodology, collaboration among entities involved in genomics, and addressing the ethical, legal, and social issues raised by genomics [12].

Apart from WHO, a few international guidelines are available that regulate medication use, including those established by the Clinical Pharmacogenetics Implementation Consortium (CPIC®). The CPIC is a globally recognized association that plays a pivotal role in the field of PGx. It was established in 2009 as a shared project between the Pharmacogenomics Research Network (PGRN), the Pharmacogenomics Knowledgebase (PharmGKB), and experts in PGx. The CPIC’s goal is to address the barrier of the difficulty in translating genetic data into clinical decisions by developing, curating, and publishing accessible, peer-reviewed, evidence-based, updatable, and detailed gene/drug clinical practice guidelines. These guidelines assist healthcare providers in understanding how genetic test results should be used to optimize drug therapy rather than whether testing is indicated [13].

### Existing regulations on PGx practice in the USA

The Food and Drug Administration (FDA) is responsible for protecting and promoting public health through proper supervision and control of medications and all related health services. The FDA has issued and developed guidance documents, warnings, guidelines, recommendations, or other regulations that may influence the practice of PGx in clinical, research, and industrial settings, as well as to address the barriers or any concerns associated with PGx implementation. Furthermore, the FDA (1) issued guidance documents on PGx and genetic tests for heritable markers, (2) listed a table of PGx biomarkers in drug labeling, (3) authorized the first direct-to-consumer test for detecting genetic variants that may be associated with medication metabolism, and (4) issued a safety communication. Additionally, the FDA also warns against the use of many genetic tests with unapproved claims to predict patient response to specific medications and takes action to advance the development of PGx [14].

Another body, the Centers for Medicare & Medicaid Services (CMS), which is a federal agency, certifies and regulates all non-research laboratory testing performed on humans through the Clinical Laboratory Improvement

Amendments (CLIA). In the context of PGx testing, any laboratory can develop/implement a PGx test; however, if the test results are to be clinically used, the test must be done in a CLIA-certified laboratory [15]. Medicare, the national health insurance program of the US government, determines and provides coverage for medically necessary PGx tests, including HLA-B*15:02 and other alleles [16]. Furthermore, in 2008, the US federal government enacted the “Genetic Information Nondiscrimination Act (GINA),” which prohibits discrimination on the basis of genetic information with respect to health insurance and employment [17].

In 2015, the American Society of Health-System Pharmacists (ASHP), a professional organization, issued a position statement on the pharmacist’s role in clinical PGx. ASHP promotes PGx education for pharmacists and supports the incorporation of PGx and its clinical application into college curricula and the Board of Pharmacy Specialties Certification Programs because it believes pharmacists have the responsibility to play a major significant role in the clinical application of PGx. This statement provides a guide on pharmacists’ responsibilities in the field of PGx and their participation in health policy development and informed recommendations for pharmacists’ functions in PGx’s clinical application [18]. Moreover, the National Human Genome Research Institute (NHGRI) provided specific genomic/PGx competencies for all healthcare disciplines to define and describe the practice-based competencies that a healthcare professional needs to specifically perform in genomic/PGx healthcare [19].

### Existing regulations on PGx practice in Canada

Health Canada (HC) is a government department in Canada that is responsible for national health policy and evaluates the safety, efficacy, and quality of health products available in the country. To date, the department has successfully implemented several promising initiatives, one of which is the Drug Safety and Effectiveness Network [20]. Working with a similar goal is the Canadian Pharmacogenomics Network for Drug Safety (CPNDS). A consortium of multicenter active surveillance and pharmacogenomics, it was established in 2004 with the aim of explaining the genetic basis of drug response and creating clinical PGx implementation tools to enhance the safety and efficacy of medications administered to children and adults [13].

### Existing regulations on PGx practice in Europe

Despite the availability of the European Medicines Institution (EMA), an agency of the European Union responsible for evaluating and monitoring of medicinal products, regulations for PGx testing tend to differ across European countries [4]. Nevertheless, the EMA has issued a number of guidelines and policies regarding PGx: a guideline on good PGx practice, which provides guidance on methods of evaluating genetic differences related to pharmacokinetics and response, where this may alter the efficacy and/or safety of a drug [21]; and a guideline on the use of PGx methods in the pharmacokinetic evaluation of medicinal products aimed to explain the requirements for the use of PGx in the pharmacokinetic evaluation of medicinal products [22]. In addition, the guideline on the key aspects of the use of PGx in the pharmacovigilance of medications addresses the influence of PGx on pharmacovigilance activities, including considerations on how to evaluate pharmacovigilance-related issues for medications with PGx associations and how to translate the results of these evaluations into optimal treatment recommendations in the labeling. Additionally, ethics policies govern the practice of PGx in research and clinical settings, such as the policy regarding confidentiality principle and the policy regarding storage of data [23].

The European Ubiquitous Pharmacogenetics Consortium (U-PGx) was established in 2016 with the aims of addressing the barriers to incorporating PGx testing into patient care while considering the variations in healthcare systems and populations across Europe. Furthermore, the U-PGx has initiated a pre-emptive PGx testing approach, whereby it notifies healthcare providers via electronic clinical decision support systems whenever a drug is prescribed or delivered to a patient with a genotype at risk [24]. As an example of European countries, the Dutch Pharmacogenetics Working Group (DPWG) was established in the Netherlands by the Royal Dutch Pharmacist’s Association in 2005. It was established with the aim of developing PGx-based therapeutic recommendations and assisting healthcare professionals in integrating these recommendations into the healthcare systems [25]. In addition, all Dutch citizens are covered by the basic level of healthcare insurance that includes PGx tests for investigation of suspected adverse drug events (ADEs) causes [26].

### Existing regulations on PGx practice in the UK

In the UK, the National Health Service (NHS), a publicly funded healthcare system, has integrated PGx into routine clinical practice with the goal of (1) optimizing medication safety and efficacy while reducing wastage of resources, including time and money on treatments and (2) issuance of guidance for using PGx information in clinical practice. In fact, the NHS established a PGx working group to identify the specific pharmacogenes and variants that should be incorporated into the National Genomic Test Directory and provided tailored clinical PGx recommendations for the UK NHS [27].

In addition, an advisory committee on genetic testing was founded to advise UK health authorities on the ethical, social, and scientific aspects of genetic testing, as well as the regulations that must be followed by providers of genetic testing services. It also evaluated the use (or potential use of) tests in clinical practice and those provided directly to the general public [28]. Recently, his Majesty’s Government (HM Government) has released two policy papers. The first is “Genome UK 2020: the Future of Healthcare,” which is a strategy outlining a vision for extending the United Kingdom’s leadership in genomic healthcare and research. The second policy paper is titled “Genome UK: 2021 to 2022 implementation plan” and outlines, through a series of commitments, how the government would advance the vision outlined in Genome UK. The current UK prescription guidelines only mandate PGx testing prior to therapy initiation for Abacavir and CBZ in patient-specific categories [29, 30].

Furthermore, two position statements were issued in 2022: one by the Royal College of Physicians and British Pharmacological Society and the other by the Royal Pharmaceutical Society. Both statements focused on using PGx to improve patient outcomes and considered the barriers and potential opportunities and benefits associated with implementing PGx testing [27, 31].

In January 2025, seven new Centers of Excellence for Regulatory Science and Innovation (CERSIs) were allocated funding of up to £1 million each to advance safer and quicker paths for innovative treatments and devices. The centers will receive funding from Innovate UK in collaboration with the Medicines and Healthcare Products Regulatory Agency (MHRA), the Office for Life Sciences, and the Medical Research Council. One of the seven centers is the Center for Excellence in Regulatory Science and Innovation in PGx, led by Liverpool University, and aims to develop guidelines for using PGx in practice and to attract more investment in this field. The CERSI in PGx will concentrate on formulating and creating guidelines, establishing industrial pathways for PGx testing, developing educational and training platforms to enhance the skills of regulators, industry, and healthcare practitioners, and conducting a broad evaluation of the health economics associated with PGx. Furthermore, it will include a patient and public work package to guarantee patient involvement in future paths [32, 33] .

### Existing regulations on PGx practice in Australia

In 2016, the Australian Genomics Health Alliance was established with the support of the National Health and Medical Research Council (NHMRC), which provided a grant of $25 million. As genomic medicine becomes the standard of care in Australia, it was established to help bridge the gap between fundamental genomic research and its clinical application by generating evidence to inform national health policy [34]. The following year, the Australian Health Ministers’ Advisory Council (AHMAC) Hospitals Principal Committee established “The National Health Genomics Policy Framework” with input from a Jurisdictional Advisory Committee. The framework is founded on the following principles: (1) the application of genomic knowledge is ethically, legally, and socially responsible, and community trust is promoted, (2) access and equity for vulnerable populations are promoted, and (3) the application of genomic knowledge for health care is supported and informed by evidence and research. Under each principle, a number of action priorities have been established to facilitate the integration of genomics into the Australian health system. For example, since 2017, it has been mandatory for Australian laboratories conducting medical tests to be accredited in accordance with Australian standards and to have systems that can ensure data transmission and storage protection [35].

The Royal College of Pathologists of Australia issued a position statement in 2018 regarding using PGx in healthcare. The purpose of the statement is to provide stakeholders, including policymakers and healthcare providers, with an update on the current status and future prospects of PGx in healthcare [36].

### Existing regulations on PGx practice in Asia

The Department of Health (DH) in Hong Kong is responsible for healthcare policy and providing essential healthcare services. The DH regulates the handling of genetic information by external entities (i.e., those not directly authorized for data processing) and is primarily governed by two existing regulations, namely the Disability Discrimination Ordinance (DDO) (Cap. 487) and the Personal Data (Privacy) Ordinance (PD(P)O) (Cap. 486). Both regulations protect patient privacy and confidentiality with regard to genetic discrimination [37].

In Japan, the Pharmaceuticals and Medical Devices Agency (PMDA) is responsible for reviewing and approving pharmaceuticals and medical devices. The agency has approved and included PGx information in the package inserts of several drugs marketed. Additionally, Japan has a National Health Insurance (NHI) system that covers the cost of genetic testing for some approved medications. The allele frequencies vary throughout the Japanese populations, where the HLA-A*31:01 allele, in particular, was found to be associated with serious CBZ-induced cutaneous adverse reactions, including SJS and TEN, but not the HLA-B*15:02 allele [38].

Interestingly, in Taiwan, a significant clinical trial conducted supported the value of HLA-B*1502 testing in preventing CBZ-induced SJS-TEN by using HLA-B*1502 screening. The findings confirmed that PGx can help identify prospectively those at genetic risk for the syndrome. Since 2010, the Taiwanese government, the Ministry of Health and Welfare (MOHW), has covered and reimbursed the costs associated with HLA-B*1502 testing [39].

To date, Singapore and Thailand are the only two Southeast Asian countries where PGx implementation has been effectively implemented at the national level due to the availability of health policies that control and regulate the clinical practice of the PGx [40]. To date, Singapore has established a standard for the provision of precision medicine that integrates the ethical, social, and legal context of this provision in healthcare and genetic data. Due to the huge impact of PGx seen, in 2013, the Ministry of Health (MOH) of Singapore issued a statement establishing screening for the HLA-B*15:02 allele as the standard of care prior to initiating CBZ treatment in new patients of Asian ancestry [41].

In Thailand, early economic studies in the Thai population reported that HLA-B*15:02-guided CBZ treatment is cost-effective compared to traditional treatment, has a high cost-benefit ratio, and reduces CBZ-induced severe adverse reactions. Consequently, HLA-B*15:02 testing is covered by health insurance [42] and has been established as a standard of treatment by the Thai government [39]. Furthermore, the Ministry of Health in Thailand introduced a “pharmacogenomics” wallet purple card with the aim of (1) highlighting patients’ *HLA* gene variant information (2) predicting the risk of developing SJS/TEN from particular drugs, and (3) meeting patients’ needs based on the medications prescribed [43].

In Malaysia, despite the fact that there are already laws that govern the regulation of PGx, the majority are relatively general and do not address the specific use and applicability of genome profiles in detail. Furthermore, several challenges exist where certain issues, like genetic discrimination and authorized personnel to order or conduct genetic testing, have not been addressed. The following are examples of the relevant existing laws and acts: (1) Medical Act 1971 (Act 50) and related medical regulations, (2) Human Tissue Act 1974, (3) Biosafety Act 2007 (Act 678), (4) DNA Identification Act 2009 (Act 699), (5) Financial Services Act 2013 (Act 758), and (6) Personal Data Protection Act 2010 (Act 709). Furthermore, a number of the aforementioned laws, such as the Human Tissue Act of 1974, is outdated and requires a revamp to fit the current scenario. Meantime, there is no law in place to regulate biobanks and genetic databases, including the use of leftover specimens and archived tissues for future examination. Likewise, there is a need for the Financial Services Act of 2013 to address genetic discrimination, particularly to ensure that insurance providers do not reject coverage to those in need [44].

Additionally, the available national guideline on medical genetics services and/or genetic testing issued by the Ministry of Health in 2019 only provides limited ethical oversights on the use of genetics in healthcare [43]. An example is the “Guideline on Ethical Issues in the Provision of Medical Genetics Services in Malaysia,” which provided ethical principles applicable to genetic services and genetic counseling, as well as laying the foundation for ethical guidelines for (1) genetic screening and testing, (2) pre-symptomatic and susceptibility testing, (3) prenatal diagnosis, (4) access to banked DNA and (5) autonomy, informed consent, disclosure, and confidentiality [45].

### Existing regulations on PGx practice in Africa

The policy governing PGx practice varies within Africa, particularly in terms of the protection of patient data. In fact, policymakers have started to recommend population-specific treatment guidelines using PGx data. Some notable examples include (1) the Data Protection Act of Mauritius, which adheres to current international standards; (2) the Protection of Personal Information Act (POPI Act) in South Africa; and (3) the Data Protection Bill of 2018 in Kenya [46]. In 2020, the Egyptian Parliament in Egypt made significant strides by approving two crucial laws that have the potential to greatly enhance genetics research and genetics services. These are the data protection law and the clinical medical research law [47].

### PGx practice policy in the context of CBZ therapy

Table 2 illustrates the comparison of policies and guidelines pertaining to applying PGx in the context of CBZ therapy. The included countries are organized alphabetically, in which the PGx testing labeling levels are categorized into “required” or “recommended,” along with information of the type of medical practice and the corresponding labeling information.

**Table 2:** Countries comparison for PGx policy in the context of CBZ therapy.

Country	Regulatory Authority	Research Consortium	Level of PGx Testing Labeling	Types of Medical Practice ^a^	Labeling information
**Australia**	Department of Health & Aged Care	–	Recommended	NK	Recommended testing for HLA-B*15:02 and HLA-A*31:01 alleles before CBZ treatment.
**Canada**	HC	CPNDS	Recommended	NK	Recommended genetic testing for all CBZ-naïve patients before treatment for HLA-B*15:02 and HLA-A*31:01 alleles.
**Europe**	EMA	U-PGx/ DPWG	Recommended	NK	Recommended testing for HLA-B*15:02, HLA-A*31:01, and HLA-B*15:11alleles before CBZ treatment.
**Hong Kong**	DH (Hong Kong)	–	Required	Standard of care	Patients should be screened for HLA-B*15:02 allele before CBZ treatment.
**Japan**	PMDA	–	Required	Standard of care	Patients should be screened for HLA-A*31:01 allele before CBZ treatment.
**Malaysia**	Malaysia MOH	–	Required	NK	Patients should be screened for HLA-B*15:02 before CBZ treatment.
**Singapore**	Singapore MOH	–	Required	Standard of care	Patients should be screened for HLA-B*15:02 before CBZ treatment.
**Taiwan**	MOHW	–	Recommended	NK	Recommended testing for HLA-B*15:02 before CBZ treatment.
**Thailand**	Thailand MOH	–	Required	Standard of care	Patients should be screened for HLA-B*15:02 before CBZ treatment.
**UK**	NHS	–	Required	Standard of care	Patients should be screened for HLA-B*15:02 allele before CBZ treatment.
**US**	FDA	–	Required	Standard of care	Black Box Warning ^b^: patients should be screened for HLA-B*15:02 allele before CBZ treatment.

Keys: NK: Not Known/Accessible to authors.

**–:** No information is available/applicable.

*CPNDS* Canadian Pharmacogenomics Network for Drug Safety, *DH* Department of Health, *DPWG* Dutch Pharmacogenetics Working Group, *EMA* European Medicines Institution, *FDA* the US Food and Drug Administration, *HC* Health Canada, *MOH* Ministry of Health, *MOHW* Taiwanese Ministry of Health and Welfare, *NHS* National Health Service, *PMDA* Pharmaceuticals and Medical Devices Agency, *U-PGx* European Ubiquitous Pharmacogenetics Consortium.

^a^Types of medical practice are either standard of care or elective/optional practice.

^b^Black Box Warning is the strongest FDA warning that signifies that clinical studies indicate that the drug carries a significant risk of preventable, severe, or even life-threatening adverse effects.

Although the WHO recently established a genomics advisory committee to speed up access to human genomics data and technologies, the body has yet to establish regulations regarding the PGx of any medications, including CBZ. Nevertheless, recent post-marketing adverse events reported to the WHO and CBZ manufacturers indicated a 10-fold increase in the incidence of SJS/TEN in some Asian countries due to the high prevalence of the *HLA* allele in the Asian population [9]. According to international guidelines, the CPIC guideline for the *HLA* genotype provides therapeutic recommendations that exclude the use of CBZ in CBZ-naïve patients who are positive for HLA-B*15:02 and any HLA-A*31:01 genotype (or HLA-A*31:01 genotype unknown) [7].

In 2007, the USA FDA issued a black box warning label stating that patients with ancestry in genetically at-risk populations (i.e., patients of Asian descent) should be screened for the presence of the HLA-B*15:02 allele prior to CBZ treatment due to the high risk of severe (and potentially fatal) dermatologic reactions [48]. In Canada, the CPNDS recommends genetic testing for all CBZ-naïve patients before treatment is instituted, with a moderate level of evidence for HLA-A*31:01 testing and strong to optional evidence for HLA-B*15:02 testing (i.e., based on the frequency of HLA-B*15:02 in the population, the patient originates from, and if this is known or not) [8]. EMA has issued the “Carbamazepine PGx guideline (HLA-A*31:01, HLA-B*15:02),” which provides recommendations for CBZ indication based on genetic variations (*HLA-A* or *HLA-B*) [49]. Similarly, the DPWG recommended avoiding the indication of CBZ and selecting an alternative, if possible, for patients positive for HLA-B*15:02, HLA-A*31:01 and HLA-B*15:11 [8].

In the UK, the monograph on CBZ in the British National Formerly 2022 [50] stated that pre-treatment screening for the HLA-B*15:02 allele in individuals of Han Chinese or Thai ancestry is required. Due to the increased risk of SJS, in the presence of the said allele, it was also recommended to avoid CBZ use, unless there is no other alternative.

Since CBZ significantly increases the risk of severe skin reactions, particularly SJS and TENS, the Australian Medicine Handbook, 2024 [51] recommended conducting genetic testing for HLA-B*15:02 allele prior to initiating CBZ in at-risk individuals such as those of Asian ancestry (especially Han Chinese, Thai, and Malay). Should the test results be positive, the use of CBZ should be avoided as far as possible. Testing of the HLA-A*31:01 allele should be considered to help reduce the risk of skin reactions, especially mild reactions and multi-organ hypersensitivity syndrome in at-risk individuals, including those of Northern European or Japanese ancestry.

In Japan, the PMDA-approved package insert for CBZ has been modified to incorporate more information regarding the HLA-A*31:01 allele [38], while Taiwan recommended using the HLA-B*15:02 testing to prevent CBZ-induced SJS-TEN [39]. In Singapore, the MOH considered screening for the HLA-B*15:02 allele before initiating CBZ treatment as the standard of care. Based on their recommendation, the use of CBZ should be avoided, and alternative treatments are indicated for patients who are HLA-B*15:02 positive [41]. Similar policy conditions and statements were established in Thailand, Hong Kong, and several centers in China [42].

In 2017, the Malaysian Society of Neurosciences recommended the following: (1) for specific patient populations, particularly Han Chinese and Malays, screening for HLA-B*15:02 is recommended prior to initiating CBZ. CBZ should not be administered to patients who test positive for HLA-B*15:02 unless the anticipated benefit outweighs the risk of SJS/TEN. Nevertheless, some challenges exist. For example, only several institutions in Malaysia offer HLA-B*15:02 testing, which is currently rather expensive. Additionally, the results may take up to three weeks. In addition, the guideline emphasized the necessity for national or regional laboratory support in order to provide the test reliably [9]. Finally, to our knowledge, no specific data or policies have been reported in relation to PGx and CBZ use in Africa to date.

## Discussion

To our knowledge, this is the first comprehensive policy review that examines the worldwide standing of the regulations that govern PGx practice, with a specific focus on CBZ therapy. The review emphasized the governmental organizations and international associations that provide guidance and support for the field of PGx practice. PGx testing, particularly for high-risk alleles associated with medications, is critical for optimizing medication safety and efficacy. Evidence indicates that using PGx test results can prevent 20 to 30% of ADEs and significantly reduce healthcare expenses and mortality associated with ADEs [2]. Since the regulatory measures in response to the rapid progress of the PGx area are also evolving, addressing the barrier of a lack of updated policies governing the practice of PGx is necessary. Further, the review proposed practical suggestions to promote the adoption and implementation of PGx in healthcare by policymakers and facilitate government decision-making.

The review’s findings revealed an upward trend in the adoption and incorporation of PGx into healthcare systems worldwide. These findings align with the rapid and growing development of various initiatives and research networks worldwide regarding PGx practice to improve health outcomes [52, 53]. A notable example is the Middle Eastern countries such as Egypt, Qatar, Saudi Arabia, and the United Arab Emirates, where PGx/genomics is currently a national healthcare priority [53]. For instance, the initiative of the Qatar Genome Program (QGP), which generates large-scale genomic databases for researchers by studying the genetic composition of Arab populations, including Qataris, to integrate precision medicine within the national healthcare system [54]. Therefore, it is crucial to speed up the introduction of PGx policies in the healthcare system. Successful integration of PGx into the healthcare system requires political support, national investments, and sustainable funding.

This review demonstrated that the USA has established policies and guidelines that address almost all aspects of PGx practice, including industry settings. This outcome aligns with the FDA’s proactive approach to including pharmacogenetic information in drug labeling, with over 400 marketed drugs associated with biomarkers in the USA [55]. As an illustration, the percentage of approvals for new medications that included PGx information on their labeling almost tripled from 10.3 to 28.2% between the years 2000 and 2020 [56]. Furthermore, these measures and advancements in the PGx field are supported by the US government, including the FDA; for example, President Obama launched the Precision Medicine Initiative in 2015 to generate the scientific evidence required for the clinical implementation of precision medicine [57].

Therefore, policy development should be promoted by drawing on the experience of PGx in the USA while considering the current state of PGx in each country. It is essential to raise awareness and enhance knowledge among all stakeholders about the substantial impact and benefits of PGx in healthcare to ensure successful implementation. In order to facilitate and support the integration of PGx into the healthcare system, it is proposed that scientific research organizations, regulatory and political bodies, healthcare practitioners, pharmaceutical and diagnostic corporations, and the general public should collaborate, as no single stakeholder can establish all of the essential requirements to advance precision medicine, including PGx integration into healthcare.

The comparison of countries’ PGx policies in the context of CBZ therapy showed substantial similarities, and observed differences can be attributed to the varying prevalence of *HLA-A* or *HLA-B* associated with CBZ-induced SJS/TENS and the degree of progress of PGx practice in each country. However, all the examined countries addressed and labeled the genetic variations associated with CBZ-induced SJS/ TENS. Screening for high-risk alleles, such as *HLA* alleles, prior to CBZ initiation is more cost-effective in populations with greater risk allele frequencies and an increased risk of developing SJS/TENS [6]. Therefore, policymakers should develop and update guidelines that regulate PGx testing and implementation, particularly for medications associated with high-risk alleles, for better health outcomes. This finding is consistent with a comparable review that focused on unraveling heterogeneity in PGx recommendations in oncology among major regulatory bodies and emphasizing the need for more comprehensive and harmonized guidance [58]. Similarly, the paper by Swen, Ingelman-Sundberg, and Patrinos (2020) discussed major design and implementation considerations for PGx panel testing, reflecting the broad variability in drug dosing guidance, consistent with the context of heterogeneity in PGx recommendations [59].

Although high-risk alleles associated with CBZ highlight the urgency of PGx regulation, the need for clear, evidence-based policies extends to all PGx-guided therapies. As a key pillar of precision medicine, PGx should be governed by robust guidelines to ensure safe, effective, and equitable use across healthcare systems, ultimately improving health outcomes.

This review identified several professional associations and societies that focused on the PGx competencies required by stakeholders, particularly healthcare professionals, and their roles in applying PGx in clinical settings. Therefore, a well-designed approach for all stakeholders, including healthcare practitioners practicing PGx, is required for better health outcomes [26]. A notable observation from the review process is that the inclusion of religious concerns pertaining to PGx testing and practice is lacking in nearly all of the countries examined. While the search strategy was not specifically designed to systematically identify religious or cultural issues, the absence of such content in the retrieved policy documents suggests that this area remains underexplored at the policy level. Religion might be a barrier to the adoption and application of PGx in healthcare. A recent study identified fatalism linked to religion as one of the emerging themes and emphasized the importance of making decisions in accordance with a personalized set of religious values [60]. Hence, engaging all PGx stakeholders, including patients with religious leaders, is crucial to developing a strategy that will aid in the development of trust between patients and healthcare professionals.

### Strengths and limitations

This is the first policy review in which countries’ policies governing PGx practice were included, providing a global overview and then focusing in-depth on the context of medication strongly associated with high-risk alleles, such as CBZ therapy. Furthermore, this review contributes to policy development pertaining to PGx practice by suggesting some recommendations that could facilitate this process. Nevertheless, this review is subject to a few significant limitations. First, CBZ serves as a clinically relevant example; however, the focus on a single drug may limit the generalizability of the findings. Nevertheless, CBZ was selected purposely to provide a concrete, in-depth picture of PGx testing implications and policy variation across countries, in addition to a clear justification detailed in the manuscript. The insights gained may still be transferable to other high-risk PGx drugs.

Second, the number of included research publications is limited by the constraints of the English language. Although this could have excluded non-English materials, we tried to mitigate this limitation by consulting gray literature, official websites, and reports from relevant authorities whenever possible. Third, variations in the availability of and accessibility to relevant governmental bodies across countries influenced the level of detail presented, reflecting source accessibility rather than preferential selection. Despite this, we exhausted our search strategy by exploring national and regional regulatory databases, official health ministry portals, and consortia websites.

### Future perspectives

The field of PGx holds significant promise for the advancement of precision medicine, yet several areas require further exploration and development to fully realize its potential. While the regulatory landscape for PGx varies significantly across countries, future efforts should aim to harmonize regulations to facilitate international collaboration and the global exchange of PGx data. The ethical and legal implications of PGx data usage, including genetic discrimination and data privacy concerns, need continued consideration. Future research should aim to develop robust ethical guidelines and legal frameworks that protect individuals while enabling the beneficial use of PGx information. Religious concerns corresponding to PGx practice should be addressed in future research as well. Moreover, to expand this effort beyond CBZ or HLA testing, future initiatives should target high-impact therapeutic areas such as oncology (e.g., fluoropyrimidines and thiopurines) and cardiovascular diseases (e.g., clopidogrel and warfarin), where PGx applications have already shown promise. Future research might consider adopting strategies such as multinational working groups or cross-agency comparative analyses to identify discrepancies and promote harmonization in PGx recommendations. It may also be helpful to create a global task force to guide the standardization of PGx information on drug labels and support its use in clinical practice. Collaborating with groups that develop treatment guidelines could help integrate PGx into broader clinical decision-making.

## Conclusion

This review sheds light on the current worldwide landscape of policies and standards governing the practice of PGx, specifically in the context of medication associated with high-risk alleles such as carbamazepine. It underscores the variability in regulatory approaches across countries, despite shared recognition of genetic risk factors for adverse drug reactions. While high-risk gene–drug interactions often draw the most attention, establishing clear policies for all PGx-informed therapies is essential to ensure consistent, safe, and effective implementation. The review also offers policy development recommendations in light of the field’s rapid evolution. Advancing PGx implementation will require not only evidence-based and context-sensitive policy updates but also improved education, awareness, and collaboration among all stakeholders. Strengthening international cooperation and aligning standards can further support the safe, effective, and equitable integration of PGx into global healthcare systems for improved health outcomes.

## Data Availability

All data generated or analysed during this study are included in this published article.
